# Supplementary figures and images for: Difference in pyruvic acid metabolism between neonatal and adult mouse lungs exposed to hyperoxia
Source: PLoS One. 2020 Sep 3;15(9):e0238604. doi: 10.1371/journal.pone.0238604 (PMC7470327; doi:10.1371/journal.pone.0238604)

## Slide 1
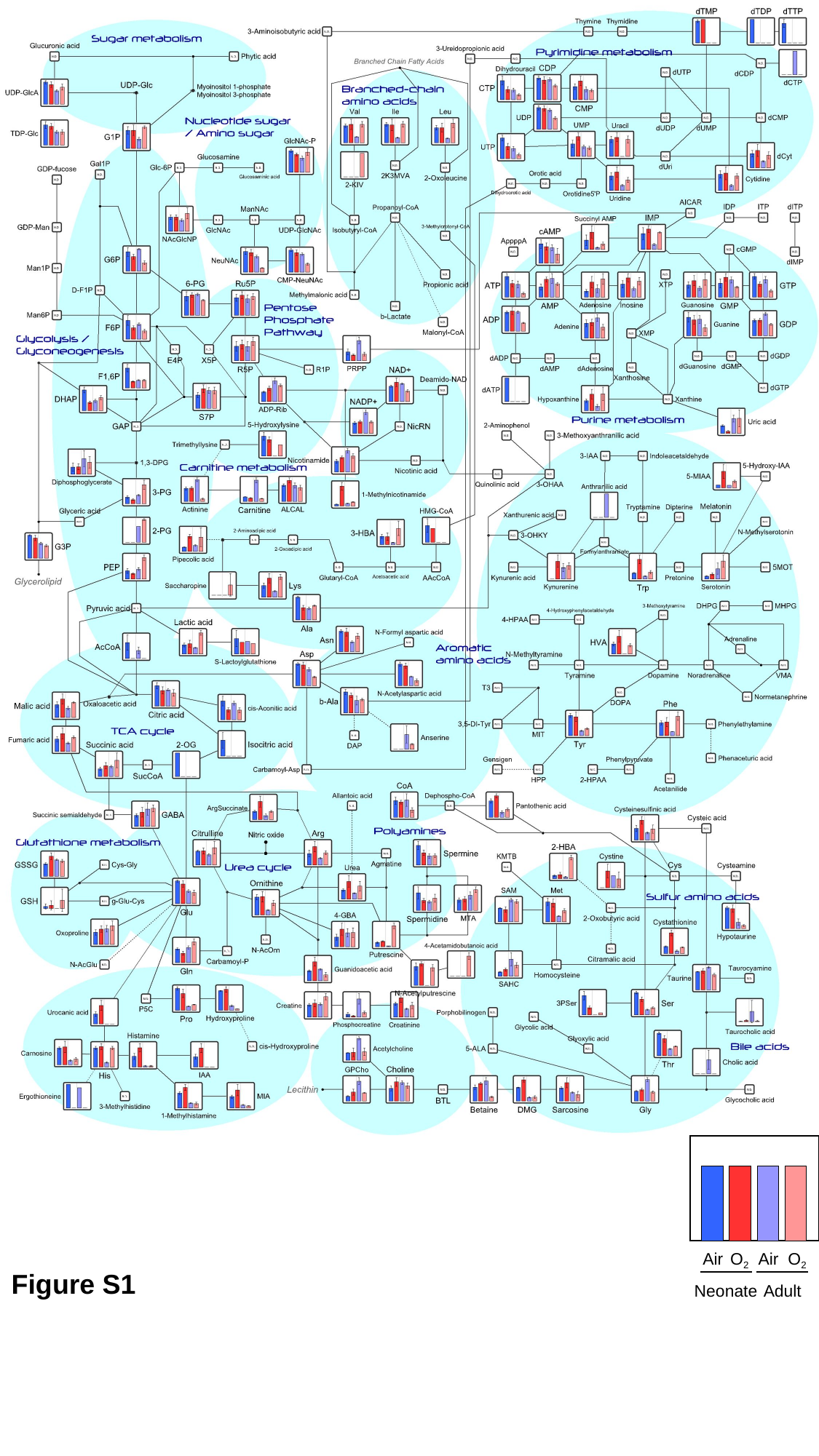

Air
O2
Air
O2
Neonate
Adult
Figure S1

Supplement: S1 Fig — For all metabolite graphs, the vertical axis shows the detected average value with standard deviation (error bar) for each comparative group, and the groups are arranged from the left in the following order: room air newborn (blue), high O2 concentration newborn (bright red), room air adult (purple), and high O2 concentration adult groups (light red). All metabolic data are presented as means ± standard deviation from biological triplicate samples. (PPTX) [file pone.0238604.s001.pptx]

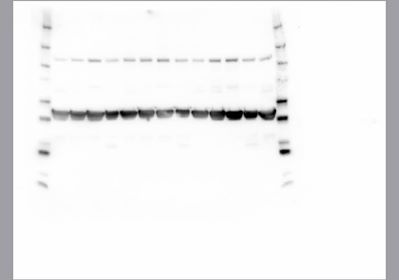

Supplement: S2 Fig — (JPG) [file pone.0238604.s002.jpg]

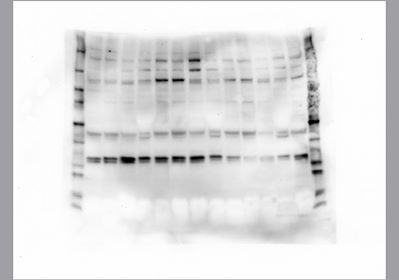

Supplement: S3 Fig — (JPG) [file pone.0238604.s003.jpg]
