# Supplementary material for: Difference in pyruvic acid metabolism between neonatal and adult mouse lungs exposed to hyperoxia
Source: PLoS One. 2020 Sep 3;15(9):e0238604. doi: 10.1371/journal.pone.0238604 (PMC7470327; doi:10.1371/journal.pone.0238604)
Supplement: Attachment — Submitted filename: Answer-to-Reviewers-Comments052520.docx [file pone.0238604.s005.docx]

Shama Ahmad, Ph.D.

Academic Editor

PLOS ONE

1160 Battery Street, Koshland Building East, Suite 225

San Francisco, CA 94111, United States

June 6, 2020

Re: Manuscript ID PONE-D-19-08318

Dear Dr. Shama Ahmad,

Please find attached a revised version of our manuscript entitled “Difference in pyruvic acid metabolism between neonatal and adult mouse lungs exposed to hyperoxia”, for publication as an Original Article in PLOS ONE.

Your comments and those of the reviewers were highly insightful and enabled us to greatly improve the quality of our manuscript. In the following pages are our point-by-point responses to each of the comments. Revisions in the text are shown using underline for additions, and ~~strikethrough~~ for deletions.

We hope that the manuscript is now suitable for publication in PLOS ONE.

We look forward to hearing from you.

Yours sincerely,

Fumihiko Namba, M.D., Ph.D.

Chief for Research

Center for Maternal, Fetal and Neonatal Medicine

Associate Professor of Pediatrics

Saitama Medical Center, Saitama Medical University

*****************************************

**Journal Requirements:**

**1. Please ensure that your manuscript meets PLOS ONE's style requirements, including those for file naming. The PLOS ONE style templates can be found at** [**http://www.journals.plos.org/plosone/s/file?id=wjVg/PLOSOne_formatting_sample_main_body.pdf**](http://www.journals.plos.org/plosone/s/file?id=wjVg/PLOSOne_formatting_sample_main_body.pdf) **and** [**http://www.journals.plos.org/plosone/s/file?id=ba62/PLOSOne_formatting_sample_title_authors_affiliations.pdf**](http://www.journals.plos.org/plosone/s/file?id=ba62/PLOSOne_formatting_sample_title_authors_affiliations.pdf)

*We edited our manuscript according to PLOSOne formatting sample provided.*

**2. At this time, we request that you please report additional details in your Methods section regarding animal care, as per our editorial guidelines: 1) Please provide details of animal welfare (e.g., shelter, food, water, environmental enrichment) 2) Please describe any steps taken to minimize animal suffering and distress, such as by administering analgesics or anesthetics, including the type and dosage. 3) Please include the method of sacrifice and 4) Please describe the post-operative care received by the animals, including the frequency of monitoring and the criteria used to assess animal health and well-being. Thank you for your attention to these requests.**

*We added details regarding animal care in our Methods section, as per your guidelines like below.*

*“All procedures and protocols were approved by the Animal Care and Use Committee of Saitama Medical University (Permit no. 1691). C57BL/6J mice (SLC, Hamamatsu, Shizuoka, Japan) for all experiments were maintained on a 12-h light, 12-h dark cycle with food and water available ad libitum. The experimental procedures were performed in accordance with the National Institutes of Health Guidelines for Laboratory Animal Care, in addition to our institutional guidelines. Neonatal pups(<12 hours old) and adult mice (8 weeks old) were randomly assigned to normoxia (room air) or hyperoxia (95% O2). Details of the hyperoxic exposure and lung tissue collection were described in our previous report [10]. Briefly, exposure to hyperoxia was conducted for 96 h in a chamber. After anesthesia with an intraperitoneal injection of pentobarbital (50 mg/kg), right lung was excised, snap-frozen with liquid nitrogen, and stored at −80°C for further analysis. All efforts were undertaken to minimize suffering.”*

**3. We note that you have included the phrase “data not shown” in your manuscript. Unfortunately, this does not meet our data sharing requirements. PLOS does not permit references to inaccessible data. We require that authors provide all relevant data within the paper, Supporting Information files, or in an acceptable, public repository. Please add a citation to support this phrase or upload the data that corresponds with these findings to a stable repository (such as Figshare or Dryad) and provide and URLs, DOIs, or accession numbers that may be used to access these data. Or, if the data are not a core part of the research being presented in your study, we ask that you remove the phrase that refers to these data.**

*We added a file of supplemental Table to support this sentence “many metabolites constituting this circuit were highly loaded in both the first and second principal components in the PCA analysis”.*

**4. Thank you for submitting the above manuscript to PLOS ONE. During our internal evaluation of the manuscript, we found significant text overlap between your submission and the following previously published works:**

**https://doi.org/10.1128/JVI.00386-17**

**https://doi.org/10.1002/cbin.10740**

**We would like to make you aware that copying extracts from previous publications, especially outside the methods section, word-for-word is not acceptable. In addition, the reproduction of text from published reports has implications for the copyright that may apply to the publications.**

**Please revise the manuscript to rephrase the duplicated text, cite your sources, and provide details as to how the current manuscript advances on previous work. Please note that further consideration is dependent on the submission of a manuscript that addresses these concerns about the overlap in text with published work.**

*We corrected text overlap between our manuscript and previous publications.*

**Reviewer(s)' Comments to Author:**

**Reviewer #1: The manuscript submitted by Kosuke and colleagues have studied the changes in lung metabolites in neonatal and adult mice exposed to hyperoxia. They found that the metabolites associated with glycolysis and gluconeogenesis were altered in the lungs of adult mice but not of the neonates after exposure to hyperoxia. This was associated with reduced PDH activity due to Pdk4 mRNA upregulation under hyperoxia. Overall, this is an interesting study, which may provide the innovative insights for different pathological responses to hyperoxic exposure between neonatal and adult mice. This reviewer has following comments, which need to addressed.**

**Comments:**

**1. N=4 mice per group is not enough.**

*We agree with Reviewer’s opinion. At least N=5 is needed for such an academic research. However, unfortunately, we used 4 samples per group because (1) this is an animal study using mice which have same background and small variation and (2) we did not have enough budget to perform metabolomics using 5 samples per group.*

**2. Protein levels of Pkd4 should be measured by Western blot to confirm the changes of its mRNA.**

*As Reviewer mentioned, we did western blot for protein levels of Pdk4 to confirm the changes of its mRNA.*

**3. It is also recommended to perform Pkd4 immunohistochemistry to show whether this is cell-specific response.**

As Reviewer recommended, Pdk4 immunohistochemistry was added.

**4. Symbols of statistical significance should be described in figure legends.**

As Reviewer mentioned, we added symbols of statistical significance in figure legends.

**5. Figure 3a is invisible.**

We moved Figure 3a to Supplementary Figure in which readers can see it by zooming on the website.

**6. The x and y axis are missing in figures 4 and 5.**

We added x and y axis in figures 4 and 5.

**7. This study described that pyruvic acid metabolism is different between neonatal and adult mice exposed to hyperoxia. This reviewer is wondering whether other metabolic pathways are also altered by hyperoxic exposure.**

We showed all metabolic pathways we investigated in Supplemental Figure. The readers can see other metabolic pathways which were altered by hyperoxic exposure.

**Reviewer #2: In the study entitled: “Difference in pyruvic acid metabolism between neonatal and adult mouse lungs exposed to hyperoxia”, Tanaka et al. have investigated underlying mechanism that explain difference between response to hyperoxia in neonatal and adults rats. Overall, the hypothesis is fine and the results are of interest. However there are some criticisms that should be answered before reconsideration.**

**Major comments**

**1. The aim of the study should be stated in a more clear way both in the abstract and at the end of the introduction. I think the novelty of the study is exploring mechanisms of different response between neonatal and adult mice and this should be clarified in the introduction. Therefore, introduction needs to be revised to be more to the point.**

We changed a statement regarding the aim of the study in a more clear way, especially in the abstract. We already mentioned the novelty of the study, “exploring mechanisms of different response between neonatal and adult mice” at the end of the introduction.

**2. The number of animals, their sex, age at the time of the study, and …. needs to be mentioned in the materials and methods section. In addition, more description of the design of the study needs to be provided.**

We added the number of animals, their sex, and age at the time of the study in the materials and methods section.

**3. I wonder if the authors have measured partial pressure of blood oxygen to assess the efficiency of the intervention. Data on this would be helpful.**

Unfortunately, partial pressure of blood oxygen was not measured. I don’t think its measurement is critical for our animal model, because hyperoxia-induced lung injury mouse model we used here and the efficiency of the intervention were widely well-known.

**4. Results of Principal component analysis (figure 2) needs to present in a more clear way. e.g. what do you mean with 24.5% in Y-axis? In addition, I think correlation matrix and whole variance that is explained using PCA needs to be mentioned.**

To show figure 2 in a more clear way as Reviewer mentioned, we added a more detailed explanation of this figure in the Figure Legend and ellipses in figure 2, which are representations of groups.

**5. Discussion section needs revision.**

**a. For example the paragraph: The effects of hyperoxic exposure on mitochondrial energy metabolism….. seems to be a reiteration of the first paragraph.**

We agree with Reviewer’s comment. We deleted this paragraph, “The effects of hyperoxic exposure on mitochondrial energy metabolism…..”

**b. In addition, some explanation about pdk isoenzymes is more appropriate for introduction rather than discussion.**

As Reviewer mentioned, we have moved some explanation about Pdk isoenzymes from discussion to introduction.

**c. This sentence: previous studies focused on the effect of hyperoxia on glycolysis and mitochondrial metabolism in adult mice, needs references.**

We added the references and the reference numbers in the text for this sentence.

**d. More comparison with similar or apposite findings of previous study should be included in the discussion.**

We added one paragraph in the discussion part about findings of previous studies which investigated the differences in oxygen tolerance between newborns and adults.

**Minor comments**

**1. Please provide some justifications for using Weltch t-test instead of usual t-test (e.g. unequal variances).**

To adopt the usual t-test in this setting, we have to confirm equality of variance. Although, performing the test for equality of variance before usual t-test arises multiplicity issues. Therefore, we chose Welch t-test which is not assuming the equality of variance.

**2. Some parts of test needs English editing. e. g. “Metabolite extraction and metabolomic analysis were”, analyses not analysis.**

Our manuscript was edited by an English editing service company before our first submission. Our revised one has also been edited by the same company and a certificate of English editing is being attached.
