# Supplementary material for: Difference in pyruvic acid metabolism between neonatal and adult mouse lungs exposed to hyperoxia
Source: PLoS One. 2020 Sep 3;15(9):e0238604. doi: 10.1371/journal.pone.0238604 (PMC7470327; doi:10.1371/journal.pone.0238604)
Supplement: Attachment — Submitted filename: Answer-to-Reviewers-Comments073020-edit-KT.docx [file pone.0238604.s006.docx]

Ch Ratnasekhar, Ph.D.

Academic Editor

PLOS ONE

1160 Battery Street, Koshland Building East, Suite 225

San Francisco, CA 94111, United States

Aug 03, 2020

Re: Manuscript ID PONE-D-20-17370

Dear Dr. Ch Ratnasekhar,

Please find attached a revised version of our manuscript entitled “Difference in pyruvic acid metabolism between neonatal and adult mouse lungs exposed to hyperoxia”, for publication as an Original Article in PLOS ONE.

Your comments and those of the reviewer were highly insightful and enabled us to greatly improve the quality of our manuscript. In the following pages are our point-by-point responses to each of the comments. Revisions in the text are shown using underline for additions, and ~~strikethrough~~ for deletions. We have deleted Ms. Kikumi Matsuoka’s name as an author and put her name in the Acknowledgement section.

We hope that the manuscript is now suitable for publication in PLOS ONE.

We look forward to hearing from you.

Yours sincerely,

Fumihiko Namba, M.D., Ph.D.

Chief for Research

Center for Maternal, Fetal and Neonatal Medicine

Associate Professor of Pediatrics

Saitama Medical Center, Saitama Medical University

**Journal Requirements:**

**1. Please ensure that your manuscript meets PLOS ONE’s style requirements, including those for file naming. The PLOS ONE style templates can be found at** [**http://www.journals.plos.org/plosone/s/file?id=wjVg/PLOSOne_formatting_sample_main_body.pdf**](http://www.journals.plos.org/plosone/s/file?id=wjVg/PLOSOne_formatting_sample_main_body.pdf) **and** [**http://www.journals.plos.org/plosone/s/file?id=ba62/PLOSOne_formatting_sample_title_authors_affiliations.pdf**](http://www.journals.plos.org/plosone/s/file?id=ba62/PLOSOne_formatting_sample_title_authors_affiliations.pdf)

*We edited our manuscript according to PLOSOne formatting sample provided.*

**2. Please ensure you have thoroughly discussed any potential limitations of this study within the Discussion section, including the limited sample size.**

*We have discussed potential limitations of this study within the Discussion section.*

**3. PLOS ONE now requires that authors provide the original uncropped and unadjusted images underlying all blot or gel results reported in a submission’s figures or Supporting Information files.**

*We have provided our original uncropped and unadjusted blot/gel image data are in Supporting Information.*

**Reviewer(s)' Comments to Author:**

**Reviewer #1: The reviewed study entitled: “Difference in pyruvic acid metabolism between neonatal and adult mouse lungs exposed to hyperoxia” by Tanaka et al. used CE TOFMS to investigate the metabolic changes between hyperoxia in neonatal and adult mice. The analytical methods are sound and very well described in the manuscript. This is an interesting study and technique and the results will provide valuable information into using neonatal mice to investigate metabolic changes associated with respiratory exposures and disease.**

**Minor Comments:**

**1. In the final paragraph of the introduction on page three, the author should include some references on using CE TOFMS as an analytical tool for metabolomics. This would validate the technique and inform the readers on the how the technique was previously used.**

*As Reviewer recommended, references that reviews CE-TOFMS technique and that is using CE TOFMS are added.*

**2. Were any of the metabolites confirmed through MS/MS analysis combined with spectral library searching, or were they identified based on intact mass and electrophoresis? If they were only identified based on these 2 parameters, the author should include some information on the mass accuracy of the instrumentation used for the analysis and the mass error for the metabolite in parts per million (ppm).**

*We did not perform MS/MS. We identified metabolite based on intact mass and migration time in capillary electrophoresis. We assigned metabolite names by matching with the library created by measurement of reference material*. *Mass error was limited within ±10ppm of theoretical value and migration tome was limited within ±0.5 minutes of the library.*
